# Mindfulness-based interventions for improving mental health of frontline healthcare professionals during the COVID-19 pandemic: a systematic review

**DOI:** 10.1186/s13643-024-02574-5

**Published:** 2024-06-20

**Authors:** Gemma Benavides-Gil, Fermín Martínez-Zaragoza, Jordi Fernández-Castro, Alicia Sánchez-Pérez, Rosa García-Sierra

**Affiliations:** 1 Department of Behavioral Sciences and Health, University Miguel Hernández, Campus de San Juan. Edf. Muhammad Al-Safra, Ctra. Alicante-Valencia, km. 87, 03550 Alicante, San Juan de Alicante Spain; 2 Department of Behavioral Sciences and Health, University Miguel Hernández, Campus de Elche. Edf. Altamira, Avda. de la Universidad, s/n, Elche, Alicante 03202 Spain; 3 Departament de Psicologia Bàsica, Evolutiva i de l’Educació, Facultat de Psicologia, Universitat Autònoma de Barcelona, Campus de Bellaterra, Barcelona, Cerdanyola del Vallès 08193 Spain; 4 Department of Pathology and Surgery, Being + Doing & Becoming Occupational Research Group (B+D+b), Institute for Health and Biomedical Research (ISABIAL), University Miguel Hernández, Campus de San Juan. Edf. Muhammad Al-Safra, Ctra. Alicante-Valencia, km. 87, 03550 Alicante, San Juan de Alicante Spain; 5 Unit Metropolitana Nord, Foundation University Institute for Primary Health Care Research Jordi Gol i Gurina (IDIAPJGol), 08303 Mataró, Barcelona, Spain

**Keywords:** Mindfulness, Mindfulness-based intervention, COVID-19, Health care professionals, Mental health, Stress, Burnout, Anxiety, Depression, Systematic review

## Abstract

**Background:**

Mindfulness-based interventions (MBIs) appear to be effective for improving the mental health of healthcare professionals (HCPs). However, the effectiveness of MBIs on extreme psychological trauma caused by the coronavirus disease 2019 (COVID-19) pandemic is largely unknown. The aim of this paper was to systematically review empirical studies of MBIs for HCPs carried out during the COVID-19 pandemic, to evaluate them and their effectiveness in different areas of mental health.

**Methods:**

The electronic databases searched were Web of Science, Scopus, PubMed, and PsycINFO. The date when each database was last searched was September 15, 2023. Randomized controlled trials (RCTs), non-randomized controlled trials (NRCTs), and non-randomized non-controlled trials (NRNCTs) focused on MBIs for health care staff who were working in healthcare centers during the COVID-19 pandemic were included. All of them employed standardized measures of mental health. The review followed the best practices and reported using PRISMA guidelines. A data collection form, adapted from the Cochrane handbook for systematic reviews of interventions, was used to extract and synthesize the results. The methods used to assess the risk of bias in the included studies were the Cochrane Risk of Bias Tool and the ROBINS-I Tool.

**Results:**

Twenty-eight studies were included in the systematic review. Overall, the methodological quality of the studies was moderate. The results showed the effectiveness of MBIs in improving levels of stress, mindfulness, and mental well-being. However, no conclusive results were found regarding the effectiveness of MBIs in improving the levels of burnout, anxiety, depression, sleep quality, and resilience of HCPs.

**Conclusions:**

The MBIs for HCPs carried out during the COVID-19 pandemic have mainly contributed to improving stress, mindfulness, and mental well-being at a time of serious health emergency. However, more robust studies at a methodological level would have been desirable.

**Systematic review registration:**

PROSPERO CRD42021267621

**Supplementary Information:**

The online version contains supplementary material available at 10.1186/s13643-024-02574-5.

## Background

The pandemic caused by the coronavirus disease 2019 (COVID-19) has been the greatest global health challenge in recent times [1]. Throughout the different waves caused by this new virus, health care professionals (HCPs) have been working for long days with high levels of pressure in unprecedented situations, characterized by serious and traumatic illness, the death of patients and colleagues, and important ethical dilemmas [2, 3]. As a result, they have largely suffered from the consequences of such stress. Numerous studies show the presence of symptoms that are commonly found in this group during the pandemic, such as stress, burnout, anxiety, insomnia, depression, and post-traumatic stress, among others [4–11].

In this scenario, various intervention strategies have been carried out to provide psychological support to HCPs, to alleviate and prevent the onset of emotional disorders. Psychological interventions, mainly focused on stress control and increasing resilience, and applied through online platforms, due to the need for social distancing, have been the most widely used during this pandemic period [12]. Some of these interventions are based on the practice of mindfulness.

Various studies show that the practice of mindfulness at work contributes to promoting the well-being of workers [13–15]. In the guide Managing work-related psychosocial risks during the COVID-19 pandemic published by the International Labour Organization [16], it is recognized that in workplaces where adequate psychological support is provided, workers can recover more quickly from stress and other mental health problems, and the proposed measures include meditation-based interventions. The usefulness of mindfulness-based interventions (MBIs) in reducing perceived stress and various psychopathological symptoms in HCPs has been demonstrated in different studies [15, 17–21].

MBIs focus on paying full attention to internal experience (sensations, emotions, and/or thoughts) with curiosity and acceptance and without judging or trying to eliminate/modify that experience. It is a process that implies attention, intention, and an open and nonjudgmental attitude [22], in other words, full awareness of the present moment. It supposes the connection with the “here and now” and being aware of what we are feeling, thinking, and doing, which helps to appreciate every moment of life. Through mindfulness, a change of perspective, “decentering” or “re-perception,” can be accomplished, so that the person can perceive internal experiences objectively and with great clarity [23, 24]. This mindfulness-enabled shift in perspective facilitates self-regulation; values clarification; cognitive, emotional, and behavioral flexibility; and the ability to deal objectively with intense emotions [22]. In this sense, by not trying to eliminate (by means of escape or avoidance mechanisms, known as “experiential avoidance”) annoying or unpleasant states, these, paradoxically, tend to dissolve more quickly, promoting feelings of calmness and serenity [25–27]. In addition, from the psychophysiological point of view, the practice of mindfulness favors the decrease in the activation of the sympathetic branch of the autonomic nervous system, the response of the hypothalamic-pituitary-adrenal axis, and cortisol levels [28].

Considering the essence of mindfulness, Good et al. [14] developed a theoretical framework to explain the mechanisms through which mindfulness improves emotional well-being in the workplace. According to these authors, its practice favors the change from cognitive processing to experiential processing. Through cognitive processing, workers evaluate and interpret external stimuli to solve problems or help make decisions in carrying out a task. However, the high demands and great uncertainty caused by the COVID-19 pandemic have in many cases exceeded the personal resources of health workers, causing repetitive and ruminative cognitive processing, which facilitates the development of anxiety, chronic stress, and automatic fear responses [29]. The practice of mindfulness could help promote awareness of the present moment, and, in this state, experiential processing could exceed cognitive processing. Experiential processing refers to the ability to direct attention to stimuli, both external and internal (emotional, physiological, cognitive) as they occur, as part of the continuous flow of consciousness, without rushing to give them meaning (meaning that usually entails a judgment of anticipation of threats). All this could contribute to the reduction of negative emotional states and the development of hedonic well-being [30].

Although it seems that MBIs are effective interventions that improve the well-being of HCPs, it is especially valuable to collect evidence of their effectiveness in a health situation as extreme as the pandemic caused by COVID-19. The stress endured by healthcare systems around the world in this period leads us to question whether the MBIs that have been carried out in this sector have sufficient potential to cause significant improvements in the mental suffering of HCPs.

While there are other systematic reviews on this topic, some do not exclusively focus on mindfulness-based interventions (psychosocial interventions [31–33], music therapy [34], reiki [34], support programs for healthcare workers’ families [35]), or they only include a specific group of HCPs (e.g., nurses) [34, 36], or they do not exclusively focus on interventions implemented during the COVID-19 pandemic but rather on previous years [31, 36–38].

In this sense, the aim of this paper is to carry out a systematic review on the effectiveness of MBIs applied during the COVID-19 pandemic in healthcare contexts to improve the mental health of HCPs and more specifically, to evaluate the content of the MBIs, the mental health areas evaluated, the instruments used for this purpose, and the effectiveness of the MBIs in each of these areas, considering experimental studies (RCTs, NRCTs, and NRNCTs). It could be stated that the main contribution of the present systematic review that differentiates it from other studies on the subject is that it focuses specifically on MBIs (and not on other types of interventions), aimed at different types of health professionals (and not only a group in particular), and carried out exclusively during the first waves of the COVID-19 pandemic (not before or after). Furthermore, special emphasis is placed on the specific content of the MBIs of each study, the various mental health variables analyzed in HCPs (stress, burnout, anxiety, sleep problems, depressive symptoms, post-traumatic symptoms, etc.), the scientific rigor of the instruments used to evaluate these variables, and the areas of mental health in which MBIs have shown greater effectiveness.

## Methods

This systematic review has been carried out following the basis of the Preferred Reporting Items for Systematic reviews and Meta-Analyses (PRISMA) 2020 Statement [39]. In Additional file 1, the PRISMA 2020 Checklist can be consulted. The review protocol was registered in the International Prospective Register of Systematic Reviews (PROSPERO) database with the reference number CRD42021267621.

### Eligibility criteria

The review question using the Population-Intervention-Comparison-Outcome-Study design (PICOS) format [40] was as follows: In HCPs working during the COVID-19 pandemic, what was the effect of MBIs on their mental health?

### Population

The types of participants were HCPs (nurses, physicians, nurse assistants, physician assistants, and other health care workers) who were working in healthcare centers (healthcare systems, hospitals, medical centers, primary care centers, mental health centers, nursing homes, home-care settings, or any other center where healthcare is provided) in any country in the world during the COVID-19 pandemic. Those studies in which the HCPs were not in direct patient care were excluded.

### Intervention

The types of interventions were those that included mindfulness in its different modalities (transcendental meditation, mindfulness focused on breathing, on thoughts, on emotions, on sounds or external stimuli, body-scan, compassion, self-compassion, heartfulness meditation, etc.), that is, programs based on mindfulness aimed at promoting full attention or awareness of the present moment with acceptance and without judgment or resistance. Considering the emergency caused by the COVID-19 pandemic, no limits were established regarding the duration, number of sessions, or application modality (in person or online) of the MBIs.

### Comparison

Studies with or without a control group (CG) have been considered. The participants in the CGs had to be integrated, as in the intervention groups (IGs), by HCPs who were working in healthcare contexts during the COVID-19 pandemic. Both passive CGs (without intervention) and active CGs (to which an intervention other than the MBIs had been applied) have been included.

### Outcomes

Studies that analyzed the effectiveness of MBIs in improving the mental health of HCPs have been included. Specifically, as secondary outcomes, the studies that analyzed, using standardized psychometric instruments, the effectiveness of MBIs (through the significant differences between pre- and post-intervention and/or between intervention groups and control groups) in improving levels of stress, burnout, anxiety, depression, sleep disturbances, post-traumatic stress, fear of COVID-19, loneliness, mental well-being, resilience, empathy, mindfulness, self-compassion, compassion, compassion satisfaction, self-efficacy, work engagement, satisfaction with life, and quality of life have been considered. Those studies that did not use standardized psychometric instruments for the evaluation of these outcomes were excluded, to guarantee the rigor of the data and to facilitate the comparison of results between studies.

### Study design

The types of studies included in this review are randomized controlled trials (RCTs), non-randomized controlled trials (NRCTs), and non-randomized non-controlled trials (NRNCTs) (single-arm before-after studies). Experimental studies have been chosen to assess the effectiveness of MBIs in improving the mental health of HCPs.

Regarding the exclusion criteria, studies that were proposals for MBI protocols that had not been implemented, pre-prints or papers that were not subjected to a peer review process, studies that only provided information on the feasibility of the intervention or levels of satisfaction with the program, and studies in which the evaluated interventions started before the COVID-19 pandemic (conducted before 2020) have not been included in the systematic review.

### Information sources and search strategy

The electronic databases used were Web of Science, Scopus, PubMed, and PsycINFO. The Cochrane Database of Systematic Reviews and PROSPERO were checked before starting the search strategies to ensure that there were no similar reviews published or similar protocol registered. The different search strategies combined the following Medical Subject Headings (MeSH) terms: Mindfulness, Meditation, Breathing Exercises, Self-Compassion, Health Personnel, Medical Staff, Nurses, Nursing Staff, Physicians, COVID-19, and SARS-CoV-2. Other related non-MeSH terms were also used. These keywords were combined with the Boolean operators AND/OR. The detailed search protocol for the different databases can be found in Additional file 2. Articles published up to September 15, 2023, were extracted. No language restrictions were applied in the searches.

### Study screening and selection

Mendeley Reference Manager was used to store the records retrieved from the database searches. The first step was to automatically remove duplicates in Mendeley. Subsequently, the results were evaluated in two rounds. The first round focused on the selection by titles and abstracts. In this round, an ad hoc form was elaborated, in which each study was identified, whether it passed to the next round, and the reasons for rejection, considering the inclusion and exclusion criteria. All studies were reviewed by two researchers (R.G.S., G.B.G.), who worked independently. Both forms were then compared, and, in cases of disagreement, a third researcher was consulted to make the final decision. There was agreement between the two first researchers in 88% of the cases. In a second round, the eligibility of the studies was evaluated through the full text, recording the specific reasons for exclusion in the same way that in the first round, although in this case the independent results obtained by the two researchers were discussed to decide eligibility by consensus. When disagreements arose between the two reviewers, they were discussed with a third researcher. The new PRISMA 2020 flow diagram for systematic reviews [39] was followed to report the number of records identified from each database and the specific reasons to exclude studies in the full-text review.

### Data extraction, evaluation, and synthesis

Data extraction was carried out using a data collection form, adapted from the Cochrane Handbook for Systematic Reviews of Interventions [41]. Two reviewers (R.G.S., G.B.G.) independently entered data from the articles included in the review. For each article, the following information was extracted in a duplicated way (see Additional file 3): general information (first author, year, article title, and country), characteristics of studies (objectives, study design, recruitment period, setting, population, sample size, and sample characteristics), intervention characteristics (intervention guiding theory, delivery modality, intervention content, timing of intervention, time follow-up, outcome measures, and time points), intervention results (findings, author’s conclusion, and theory to explain the findings), and source of funding.

After this duplicated data extraction, the researchers pooled the extracted data to discuss possible differences. In cases where some data did not appear in the article or that clarification was desired, the corresponding author of the article was contacted.

A systematic review may or may not contain a meta-analysis (quantitative vs. qualitative systematic review) depending on whether the data from previous studies addressing the desired question can or cannot be combined [42]. Clinical and methodological heterogeneity of the studies led us to conclude that a systematic review with meta-analysis (quantitative review) was not possible to be performed. For this reason, a non-quantitative review or descriptive synthesis was performed. However, quantitative data (descriptive statistics) were extracted from the articles and, when possible, effect sizes and confidence intervals of effect sizes. To carry out the descriptive synthesis, two work meetings were held (G.B.G., F.M.Z.) to establish a consensus based on the data extracted from each study.

### Methodological quality assessment

The methodological quality was evaluated with the Cochrane Risk of Bias Tool [43] for the risk of bias assessment of the selected RCTs. For NRCTs and NRNCTs, the ROBINS-I tool [44] was used. Based on these guidelines, a table with all the items was done to extract and critically assess the different dimensions of bias. These dimensions are participant selection, confounding variables, classification of interventions, blinding, deviation from intended data, measurement of outcomes, and selection of the reported result. The risk of these biases has been evaluated in each article selected in a categorization of low, high, or unclear. The assessment has been done by two researchers independently (R.G.S., G.B.G), with the collaboration of a third party when no agreement was able to be reached. The form with the bias dimensions is provided in the Additional file 4.

## Results

### Study selection

**Fig. 1:**
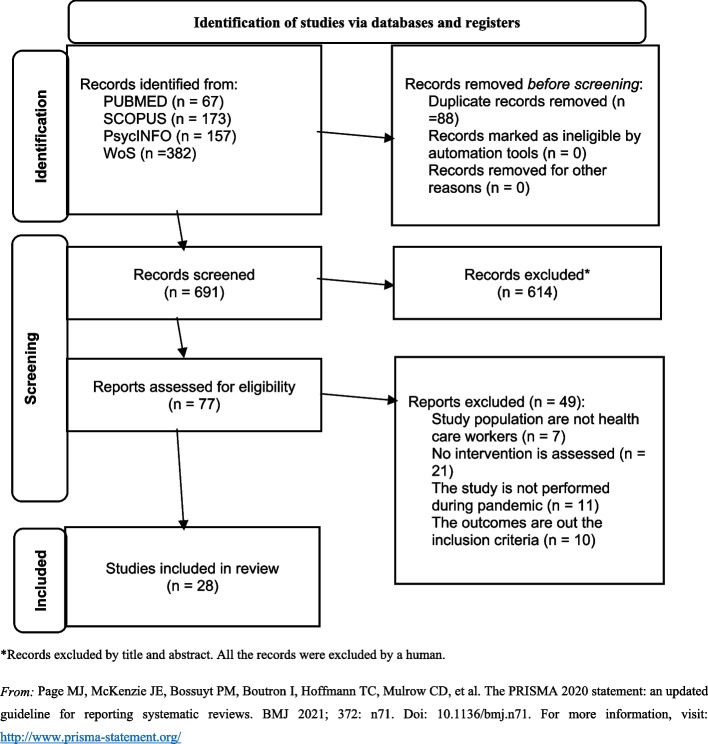
PRISMA flow diagram. *Records excluded by title and abstract. All the records were excluded by a human. *From:* Page et al. [39]. For more information, visit: http://www.prisma-statement.org/

Figure 1 shows the study selection process and results based on the PRISMA 2020 flow diagram for new systematic reviews [39]. In total, 779 articles were identified. A total of 88 of these articles were removed because they were duplicates. After the titles and abstracts were screened, 77 articles were included in the next stage. Of these, 49 articles were excluded due to different reasons. As a result, 28 studies satisfied the eligibility criteria and were included in the review. Of them, 11 were RCTs, and 17 were NRCTs and NRNCTs.

### Risk of bias

Methodological evaluation was conducted in all the articles. Studies were not excluded based on low methodological quality. Tables 1 and 2 present the results of the critical appraisal.

**Table 1:** Assessment of methodological quality of the RCTs

Bias domain	Source of bias	**Judgment**										
		AlQarni	Cao	Fiol-DeRoque	Hsieh	Keng	Li	Marotta	Nourian	Thimmapuran	Vajpeyee	Yıldırım
Selection	Random sequence generation	Low	Unclear	Low	High	Low	Low	High	Low	Low	Unclear	Low
	Allocation concealment	Low	Unclear	Low	Unclear	Low	Low	High	Low	Low	Low	Low
Performance	Blinding participants and researchers	Low	Unclear	Low	High	Low	Unclear	High	Unclear	Unclear	Unclear	Unclear
Detection	Blinding of outcome assessment	Low	Low	Low	Unclear	Low	Low	Low	Unclear	Unclear	Low	Low
Attrition	Incomplete outcome data	Low	Low	Low	Low	Low	Low	Unclear	Low	Low	Low	Low
Reporting	Selective reporting	Low	Low	Low	Low	Low	Low	Low	Low	Low	Low	Low

*Low*, low risk; *High*, high risk; *Unclear*, unclear risk

**Table 2:** Assessment of methodological quality of the NRCTs and NRNCTs

**Bias domains—source of bias**	**Judgment**															
	Al Ozairi	Azizoddin	Cepeda- López	DeTore	Divya	Franco	Gherardi-Donato	Ibrahim	Kim*	Klatt	Luton	Miyoshi	Nestor	Osman	Pandey	Prado
PI**—**confounding	High	Low	Low	Low	Low	Unclear	Low	High	High	High	Unclear	High	Low	Unclear	High	High
PI**—**selection of participants	Low	Unclear	High	High	Low	Unclear	Low	Low	High	High	High	Unclear	Unclear	Unclear	High	High
AI**—**classification of interventions	-	-	-	-	Low	High	-	Unclear	-	Low	Unclear	-	Low	-	-	-
PtI**—**deviations from intended interventions	Low	Low	Low	Low	Low	Low	Low	Low	Low	Low	Low	Unclear	Unclear	Low	Unclear	Unclear
PtI**—**missing data	Unclear	Unclear	Unclear	High	Low	Low	Unclear	Unclear	Unclear	Unclear	High	Unclear	Unclear	Unclear	Unclear	Unclear
PtI**—**measurement of outcomes	Low	Low	High	High	Low	Low	Low	Low	Unclear	Low	High	Low	Low	Unclear	Low	Low
PtI**—**selection of the reported result	Low	Low	Low	Low	Low	Low	Low	Low	Unclear	Unclear	Unclear	Low	Low	Low	Low	Low

*PI*, pre-intervention; *AI*, at intervention; *PtI*, post-intervention; *Low*, low risk; *High*, high risk; *Unclear*, unclear risk

^*^Two studies

Three of the 11 RCTs (27.3%) show a low risk of bias in all domains evaluated. This means that, in general, the studies present an adequate explanation regarding the entire research process (recruitment, group allocation, MBI, data extraction, statistical analysis, etc.). The greatest source of bias is the blinding of participants and researchers, where six articles (54.5%) present unclear risk and two (18.2%) high risk. Regarding random sequence generation, two papers (18.2%) present unclear risk and two (18.2%) high risk. And as for allocation concealment, two articles (18.2%) present unclear risk and only one (9.1%) high risk.

Based on the judgment of the risk of bias of the 17 NRCTs and NRNCTs, only one has a low risk in all its bias domains. The source of bias with higher risk is the missing data, where 75% of the studies have unclear risk because they do not explain how the missing data have been treated. The second source of bias is the selection of the participants. In this case, 43.7% of the studies have high risk and 31% of them have unclear risk, in most cases related to a poor description of the selection of the different cohorts of the studies.

### Study characteristics

The characteristics of the included studies are summarized in Table 3. Nine studies were published in 2021, 12 of them in 2022, and 7 in 2023. Eleven of the studies were RCTs (5 of them with active CGs), 5 NRCTs, and 12 NRNCTs. Two studies used a mixed method design, with quantitative and qualitative data. Nine studies included follow-up data, ranging from one to 6 months.

**Table 3:** Study characteristics

**Author/year country**	**Design**	**Setting and participants**	**Characteristics of the participants**	**Outcome measures**	**Assessment instruments**
AlQarni et al. 2023 Saudi Arabia [45]	RACT	Various hospitals. 125 HCPs (physicians, respiratory therapists, and nurses)	*Intervention group:* Age (mean ± SD): 32 ± 7 Females 42 (65.6%), males 22 (34.4%) *Control group:* Age (mean ± SD): 34 ± 8 Females 50 (82%), males 10 (16.4%)	Psychological well-being Resilience Anxiety	WHO-5 CD-RISC-10 STAI-Adults
Al Ozairi et al. 2023 Kuwait [46]	NRNCT	Healthcare system. 125 physicians	Age: 18–34 (*n* = 65, SD = 52.0), 35–44 (*n* = 42, SD = 33.6), 45–64 (*n* = 18, SD = 14.4). Females 99 (79.2%).	Anxiety Depression Mindfulness	GAD-7 PHQ-9 FFMQ
Azizoddin et al. 2022 USA [47]	NRNCT Pilot study	Two urban hospitals. 31 emergency clinicians	Mean age 41.6 (SD = 10.4). Females 19 (61%). Physicians 14 (46%), nurses 7 (22%), physician-assistants 10 (32%).	Burnout Depressive symptoms Anxiety symptoms Perceived stress Sleep difficulties	MBI PHQ-8 GAD-7 PSS-4 PROMIS Sleep Disturb. 8a
Cao et al. 2022 China [48]	RCT	General hospital. 108 nurses	*Intervention group:* Age (*n*): 18–25 (11), 26–30 (27), ≥ 31 (15). Females 49, males 4. *Control group:* Age (*n*): 18–25 (13), 26–30 (30), ≥ 31 (12) Females 48, males 7.	Mindfulness Perceived stress Burnout Psychological well-being	FFMQ CPSS MBI SPWB
Cepeda-López et al. 2023 México [49]	NRNCT	2 private hospitals. 643 nurses	Mean age 34.1 (SD = 9.22). Females 82.6%, males 17.4%.	Subjective well-being Resilience Mindfulness Perceived stress Burnout	PERMA Profiler BRS MAAS PSS BCSQ-12
DeTore et al. 2022 USA [50]	NRCT	Healthcare system. 148 HCPs	Mean age 43.32 (SD = 13.07). Females 134 (90.5%), males 13 (8.5%), others 1 (1%). Physicians 23 (15.5%), nurses 46 (31.1%), others 79 (53.4%).	Depression Anxiety Resilience Loneliness Self-compassion Burnout	PHQ-4 PHQ-4 BRS UCLA Loneliness SCS MBI
Divya et al. 2021 India [51]	NRNCT Pilot study	HCPs authorized to practice by the state. 92 HCPs	Mean age 43.1 (SD = 11.1), range 19–72. Females 54 (58.7%), males 38 (41.3%). Physicians 56 (60.8%), non-physician clinicians 17 (18.6%), non-clinicians 8 (8.7%).	Depression Anxiety Stress Sleep quality Resilience Satisfaction with life	DASS-21 DASS-21 DASS-21 PSQI CD-RISC SWLS
Fiol-DeRoque et al. 2021 Spain [52]	RACT	Hospital, primary care, and home-care settings. 482 HCPs	Mean age 41.37 (SD = 10.4). Females 401 (83%), males 81 (17%). Nurses 161 (33%), doctors 153 (32%), nurse assistants 147 (31%), others 22 (4%).	Depression Anxiety Stress Post-traumatic stress Burnout Insomnia Self-efficacy	DASS-21 DASS-21 DASS-21 DTS MBI-HSS ISI GSE
Franco et al. 2021 USA [53]	NRCT	Pediatric hospital. 48 clinical or non-clinical nurses	*Intervention group:* Mean age 46.05. Females 21, males 1. *Control group:* Mean age 38.33. Females 22, males 4.	Self-compassion Mindfulness Compassion Compassion satisfaction Burnout Post-traumatic stress Depression Anxiety Stress Resiliency Job engagement	SCS CAMS CFO ProQOL ProQOL ProQOL DASS DASS DASS SRS JES
Gherardi-Donato et al. 2023 Brazil [54]	NRNCT	Healthcare system. 44 nursing professionals (nurses, technicians, and assistants)	Mean age 33.8 (SD = 5.6). Females 37 (84.1%), males 7 (15.9%).	Perceived stress Anxiety Depression symptoms Mindfulness	PSS-14 BAI BDI-II MAAS, FFMQ
Hsieh et al. 2022 Taiwan [55]	RCT	Medical center. 79 nurses.	*Intervention group:* 40 Mean age 42.30 (SD = 8.49). *Control group:* 39 Mean age 32.51 (SD = 8.24).	Perceived stress Burnout	PSS OBI
Ibrahim et al. 2022 Indonesia [56]	NRCT	Various hospitals. 50 nurses	*Intervention group:* Mean age 35.2 (SD = 7.9), range 24–50. Females 16 (64%), males 9 (26%). *Control group:* Mean age 32.6 (SD = 7.9), range 24–50. Females 8 (32%), males 17 (68%).	Psychological well-being	WEMWBS
Keng et al. 2022 Singapore [57]	RACT	Unidentified setting. 80 HCPs	Mean age 30.18 (SD = 6.19), range 22–54. Females 72 (90%), males 8 (10%). Nurses 47 (58.75%).	Depression Anxiety Fear of COVID-19 Post-traumatic stress Subjective well-being Burnout Compassion satisfaction Perceived sleep quality Mindfulness Self-compassion	DASS-21 DASS-21 FCV-19S PCL-C PWI ProQOL ProQOL PSQI FFMQ SCS
Kim et al. 2022, 2023 [58, 59] Canada	NRNCT Mixed method	Psychiatric hospital. 130 HCPs	Age (%): −30 (17.05%), 31–50 (54.26%), +50 (28.68%) Females 93.02%.	Resilience Burnout	NMRQ MBI-HSS
Klatt et al. 2021 USA [60]	NRNCT	Medical center. 99 HCPs. *Only included the Covid-19 period cohort*	Females 84%, males 16%.	Burnout Resilience Work engagement Perceived stress	MBI CD-RISC UWES PSS
Li et al. 2022 China [61]	RCT	HCPs aiding in Wuhan. 134 medical staff	Age range 21–60. Females 70.1%. Doctors 34 (25.4%), nurses 86 (64.2%), others 14 (10.4%).	Depression Anxiety Perceived stress Insomnia	PHQ-9 GAD-7 PSS AIS
Luton et al. 2021 UK [62]	NRCT	Core surgical boot camp. 38 surgical trainees	*Intervention group:* Mean age 28, range 25–32. Females 5, males 9. *Control group:* Mean age 29, range 26–35. Females 4, males 10.	Burnout Stress Mindfulness Depression Anxiety	aMBI PSS-10 CAMSR 9PHQ-2 STAI-6
Marotta et al. 2022 Italy [63]	RCT	Two hospitals. 26 HCPs *Only included the Covid-19 period cohort*	*Intervention group:* Mean age 45.2 (SD = 15.1). Females 12 (80%), males 3 (20%). Physicians 3 (20%), nurses 9 (60%), others 3 (20%). *Control group:* Mean age 39.2 (SD = 15.1). Females 10 (90.9%), males 1 (9.1%). Physicians 2 (18.2%), nurses 7 (63.6%), others 2 (18,2%).	Psychological well-being Perceived stress Burnout Fear of COVID-19	PGWBI PSS MBI FCV-19S
Miyoshi et al. 2022 Japan [64]	NRNCT	University hospital. 13 HCPs	Mean age 49 (SD = 8.6). Females 11 (84.6%), males 2 (15.4%). Nurses 7 (53.8%), doctors 3 (23.1%), others 3 (23.1%).	Depression Burnout Stress Resilience Self-compassion Empathy	PHQ-9 MBI-HSS SOC-13 CD-RISC SCS-SF JSE
Nestor et al. 2023 USA [65]	NRCT	3 hospitals. 130 HCPs	*Intervention group:* Mean age 44.9 (SD =9.9). Females 40 (61.5%), males 25 (38.5%). Physicians 30 (46.2%), nurses 14 (21.5%), others 21 (32.2%). *Control group:* Mean age 43.6 (SD = 12.0). Females 35 (53.8%), males 30 (46.2%). Physicians 25 (38.5%), nurses 15 (23.1%), others 25 (38.4%).	Depression Anxiety Insomnia Burnout Well-being	BSI-18 BSI-18 ISI MBI-HSS (MP) WEMWBS
Nourian et al. 2021 Iran [66]	RACT	Two COVID hospital wards. 41 bachelor’s degree in nursing	Mean age 35.60 (SD = 8.21). Females 34 (59.6%), males 7 (12.3%).	Sleep quality	PSQI
Osman et al. 2021 South Africa [67]	NRNCT Mixed method	Health care system. 47 medical professionals	Mean age 34 (SD = 18). Medical doctors and trainees 46%, psychologists 16%, physiotherapists 14%, occupational therapists 14%, others 10%.	Trait mindfulness Perceived stress Burnout	MAAS PSS aMBI
Pandey et al. 2021 India [68]	NRNCT	Dental units of clinics. 30 oral healthcare professionals	Mean age 40.5 (SD = 2.5), range 30–50. Females 18 (60%), males 12 (40%).	Quality of life	WHOQOL-BREF
Prado et al. 2023 USA [69]	NRNCT	Unidentified setting. 100 health care workers	Age (*n*): 18–25 (8), 26–33 (26), 34–40 (29), 41–48 (14), 49–56 (13), 57–64 (7), 65–89 (2), No answer (1). Females 86 (86%), males 13 (13%), other 1 (1%).	Perceived stress	PSS-10
Thimmapuram et al. 2021 USA [70]	RCT	Four hospitals. 155 physicians advanced practice providers	Mean age 46 (SD = 11.08). Females 103 (66%), males 46 (30%), others 6 (4%). Attending physicians 61 (39%), resident physicians 12 (8%), certified registered nurse practitioners 58 (37%), physician assistants 18 (12%), others 6 (4%).	Loneliness Sleep quality	UCLA Loneliness PSQI
Vajpeyee et al. 2022 India [71]	RCT	Healthcare service. 209 HCPs	Age range 18–60. Females 34 (16.26%), males 175 (83.73%).	Depression Anxiety Stress	DASS-42 DASS-42 DASS-42
Yıldırım et al. 2022 Turkey [72]	RACT	University hospital. 104 nurses	*Intervention group:* Mean age 27.55 (SD = 5.24). Females 40 (77%), males 43 (83%). *Control group:* Mean age 29.11 (SD = 6.57). Females 12 (23%), males 9 (17%).	Anxiety Psychological well-being Work-related strain	STAI-I PWB WRSI

*RCT*, randomized controlled trial; *RACT*, randomized active controlled trial; *NRCT*, non-randomized controlled trial; *NRNCT*, non-randomized non-controlled trial

### Characteristics of the participants

The characteristics of the participants are summarized in Table 3. The sample sizes of participants included in the 28 studies ranged from 13 to 643, with a higher percentage of women (74%). The participants included nurses, physicians, nurse assistants, physician assistants, and other health care workers (oral healthcare professionals, occupational therapists, psychologists, physiotherapists, pharmacists, technicians, and others). The largest proportion corresponds to nursing and medical staff (90%). Seven of the studies were carried out in the USA, 3 in India, 2 in Canada, 2 in China, and 1 in each of the following countries: Brazil, Indonesia, Iran, Italy, Japan, Kuwait, México, Saudi Arabia, Singapore, South Africa, Spain, Taiwan, Turkey, and the UK. The studies had been carried out in hospitals (52%), health care systems, primary care, medical centers, home-care settings, and academic health center settings.

### Key themes

The key themes arose from the results of the articles (see Table 4), related to the characteristics of intervention (MBIs modalities, duration, sessions, in-person/virtual modality, trainer profile, follow-up…), the outcomes measures (standardized psychometric instruments used), and the MBIs effectiveness in the mental health areas (stress, burnout, anxiety, depression, sleep quality, resilience, mindfulness, mental well-being, fear of COVID-19, compassion, compassion satisfaction, self-compassion, loneliness, post-traumatic stress, work engagement, self-efficacy, satisfaction with life, quality of life, and empathy).

**Table 4:** Key themes extracted from the reviewed articles

MBIs on the mental health of HCPs	Characteristics of the interventions	Mindfulness with different meditation strategies Mindfulness combined with other techniques (psychoeducation, skills for daily life, emotional skills, yoga, music therapy, etc.)
	Outcome measures and instruments	Stress Burnout Anxiety Depression Sleep quality Resilience Mindfulness Mental well-being Others
	MBIs effectiveness	

### Characteristics of the interventions

The characteristics of the interventions are summarized in Table 5.

**Table 5:** Characteristics and results of the interventions

**Study**	**Intervention and timing**	**Main findings**	**Main findings (detailed)**
AlQarni et al. 2023 [45]	MP3 recordings via a website: Mindfulness of breath, movements, sensations, emotions, and thoughts. *Control group:* Progressive muscle relaxation. Tensing and relaxing specific muscle groups in sequence. 2 weeks. Daily 20-min sessions	Y - Psychological well-being N - Resilience M - Anxiety	Both groups showed a statistically significant improvement in mental well-being, and the percentage of improved cases was higher in group MBI (52, 81.3%) than in group relaxation (31, 50.8%), 2 = 12.9, *p* = .0001, adjusted odds ratio (OR), 95% CI = 4.19 (1.5–11.6). None of the groups showed significant improvement in resilience (Pillai’s trace F (1,105) = .246, *p* = .621). Both groups showed a statistically significant decrease in state anxiety (Pillai’s trace F (1,82) = 15.7, *p* = .0001).
Al Ozairi et al. 2023 [46]	Remote sessions (zoom): Mindful breathing, sitting meditations, body scans, sound meditations. Conscious movements and walking. Informal practices to aid compassion and kindness. 2 weeks. 8 sessions. 2 h a day. Home practice.	Y - Anxiety Y - Depression Y - Mindfulness	Significant reductions in anxiety (*p* < .001; *d* = .9) and depression (*p* < .001; *d* = .92). Significant improvement in mindfulness (*p* < .001; *d* = 1.14).
Azizoddin et al. 2022 [47]	In-person and remote sessions (zoom): Transcendental Meditation. Use of a repetitive mantra while sitting with closed eyes. 4 days. 8 sessions. 20-min home practice. *3-month follow-up.*	Y - Burnout Y - Depressive symptoms Y - Anxiety symptoms Y - Perceived stress Y - Sleep difficulties	Significant reductions at post intervention, with significant decreases at the 3-month follow-up, in burnout (emotional exhaustion: *p* = .002, *d* = .45; depersonalization: *p* < .001, *d* = .43; professional accomplishment: *p* = .036, *d* = .43), sleep disturbance (*p* < .001, *d* = .70), symptoms of depression (*p* < .001, *d* = .86), anxiety (*p* < .001, *d* = .87), and stress (*p* < .001, *d* = .75).
Cao et al. 2022 [48]	Online (Tencent Meeting): Balint group–based activities combined with MBSR (breathing, body-scan, emotions, thoughts, loving kindness, contemplation, eating, drinking…). 8 weeks. 2 sessions of 1.5 h per week. Mindfulness practice for 20 min daily.	Y - Mindfulness Y - Perceived stress Y - Burnout Y - Psychological well-being	In the IG, mindfulness (*t* = −59.82, *p* = .000) and psychological well-being (*t* = −78.93, *p* = .000) increased, and perceived stress (*t* = 53.99, *p* = .000) and burnout (*t* = 54.94, *p* = .000) decreased from pre to post. No significant changes were found for the CG.
Cepeda-López et al. 2023 [49]	Online: mindfulness-based stress reduction, single-focus meditation, self-regulation exercises, breathing practices, awareness practices, and spirituality and reframing. 12 weeks. 36 micro-practices. 3 times per week. *6-month follow-up*	N - Subjective well-being N - Resilience Y - Mindfulness Y - Perceived stress N - Burnout	Significant reductions in subjective well-being (*p* < .001; *d* = .48), resilience (*p* < .001; *d* = .25), and stress (*p* < .001; *d* = .33) from pre-test to 6-month follow-up. Significant improvement in mindfulness (*p* < .008; *d* = − .14). Burnout remained stable.
DeTore et al. 2022 [50]	Online platform (HealthStream): Resilience and mindfulness skills. Cognitive flexibility. Self-compassion. Three 12–19 min videos. *2-month follow-up.*	Y - Depression Y - Anxiety M - Resilience ND - Loneliness ND - Self-compassion ND - Burnout	No significant change in resilience from pre- to post-intervention (*t* = 1.46, *p* = .153), but yes at 2-month follow-up (*t* = 2.88*, p* = .010), and significant decreases in anxiety and depression (emotional distress) at post (*t* = 3.09*, p* = .004) and 2-month follow-up (*t* = 2.97*, p* = .009) in the IG. No significant changes in the CG.
Divya et al. 2021 [51]	Online workshop: Sudarshan Kriya Yoga (SKY). Yogic breathing exercises. 4 days. 8 h. 2 h per session. 35-min home practice. *40 days follow-up.*	M - Depression M - Anxiety M - Stress M - Sleep quality Y - Resilience Y - Satisfaction with life	Statistically significant reduction in stress (*p* < .001), anxiety (*p* = .001), depression (*p* < .001), and sleep problems (*p* < .001) immediately after the intervention, but not at 40 days. Statistically significant improvement in resilience (*p* < .001) and life satisfaction (*p* < .001) immediately after the program, and it continued to increase on day 40 (*p* = .015, *p* < .001).
Fiol-DeRoque et al. 2021 [52]	Online, app: Cognitive-behavioral therapy and mindfulness in four areas (emotional skills, healthy lifestyle behavior, work stress and burnout, and social support). *Control group:* information about mental healthcare of HCPs during the COVID-19 pandemic. 2 weeks.	N - Depression M - Anxiety M - Stress M - Post-traumatic stress N - Burnout M - Insomnia N - Self-efficacy	No significant differences in the variables between groups at post-intervention. But in the subgroup consuming psychotropic medications, the IG significantly improved anxiety (−.26 (−.45 to −.08), stress (−.30 (−.50 to −.09)), post-traumatic stress (−.20 (−.37 to −.03)), and insomnia (−.16 (−.30 to −.02)) compared to the CG, whereas no significant differences were observed in depression, burnout, and self-efficacy. In the subgroup receiving psychotherapy, the IG significantly improved anxiety (−.24 (−.48 to .00)), stress (−.27 (−.55 to .001)), and insomnia (−.20 (−.42 to .02)) compared to the CG, whereas no significant differences were observed in depression, post-traumatic stress, burnout, and self-efficacy.
Franco and Christie 2021 [53]	Face-to-face: Activities about self-compassion. Coloring supplies and small toys. Booklet describing the concepts and practices. 1 day. 6 sessions of 1 h. *3-month follow-up.*	Y - Self-compassion Y - Mindfulness M - Compassion Y - Compassion satisfaction Y - Burnout N - Post-traumatic stress N - Depression Y - Anxiety Y - Stress M - Resiliency N - Job engagement	In the IG, self-compassion (*t* (79.7) = 5.77, *p* < .001), mindfulness (*t* (77.7) = 5.64, *p* < .001), and compassion satisfaction (*t* (78) = 2.49, *p* < .05) increased from pre to post and from pre to 3-month follow-up; burnout (*t* (78) = −4.29, *p* < .001), anxiety (*t* (77.8) = − 3.47, *p* < .01) and stress (*t* (78) = −3.41, *p* < .01) decreased and this change was maintained at 3 months; and compassion (*t* (78.7) = 3.39, *p* < .01) and resiliency decompression (*t* (78.8) = 4.09, *p* < .001) showed significant increases at 3-month follow-up. No significant changes were found for the CG.
Gherardi-Donato et al. 2023 [54]	Online: Mindfulness of breathing, the body, sensations, sounds, thoughts, and emotions. 8 weeks. 8 sessions of 2 h. Informal practices.	Y - Perceived stress Y - Anxiety Y - Depression symptoms Y - Mindfulness	Significant reductions in perceived stress (*Z* = -4.25; *p* < .001), anxiety (Z = -3.16; *p* = .002) and depression (*Z* = −3.50; *p* < .001). Significant improvement in mindfulness (*Z* = 3.92; *p* < .001).
Hsieh et al. 2022 [55]	In-person: Gong meditation. Gong with real-time adjusted rhythm. 7 sessions of 50–60 min on 2 consecutive days.	Y - Perceived stress Y - Burnout	In the IG, there were significant post-intervention reductions in perceived stress (*β* = 4.20, *p* < .001) and in all burnout subscales: personal burnout (*β* = 6.53, p < .001), work-related burnout (*β* = 5.85,* p* < .001), client-related burnout (*β* = 3.98, *p* < .001) and over-commitment to work (*β* = 3.69, *p* < .001), compared to the CG.
Ibrahim et al. 2022 [56]	Online, WhatsApp: Protocol of mindfulness breathing meditation. Video practice guidelines and tutorials. 4 weeks. Twice a week. 15 min per session.	Y - Psychological well-being	Statistically significant difference in psychological well-being pre- post-intervention in the IG (*t =* −*4.56, p* = .000). No statistically significant difference pre-post in the CG.
Keng et al. 2022 [57]	Headspace app: Introduction to mindfulness practice. Mindfulness of breathing, of thoughts, of sounds. *Control group:* Daily training component (games involving problem solving, memory, and attention). 10-day basic course. One 10-min practice each day. 3-week practice period. *1-month follow-up.*	N - Depression N - Anxiety M - Fear of COVID-19 N - Post-traumatic stress N - Subjective well-being N - Burnout M - Compassion satisfaction M - Perceived sleep quality M - Mindfulness M - Self-compassion	No significant between-condition changes in any variables from pre- to post-intervention (*p* > .05). From pre- to 1-month follow-up, significantly improvements in the IG, compared to the CG, in fear of COVID-19 (*β* = −.239, *p* = .005), compassion satisfaction (*β* = .183, *p* = .007), mindfulness (*β* = .255, *p* = .002), self-compassion (*β* = .208, *p* = .005), and sleep quality (*β* = .002, *p* = .002).
Kim et al. 2022, 2023 [58, 59]	Online (zoom): Mindfulness Ambassador Program (MAP). Anchoring with breath, anchoring with external stimuli, mind–body awareness, compassion. 4 weeks. 4 sessions of 30 min. Daily practices. *1-month follow-up.*	Y - Resilience Y - Burnout	Resilience significantly increased after the mindfulness program compared to the baseline, maintaining the effect after 1 month. The emotional exhaustion level decreased significantly at the end of the intervention and at follow-up (*β* = −4.59, 95% CI = −7.39 to −1.78, *p* < .01). Depersonalization was significantly lower for those who practiced mindfulness 4–7 times/week than those who never practiced mindfulness after the session (*β* = −3.87, 95% CI = −7.17 to −.57, *p* < .05). No significant improvement in personal accomplishments.
Klatt et al. 2021 [60]	Online: Mindfulness in Motion (MIM). Explanation of the science behind mindfulness. Experiential mindfulness meditation and gentle yoga session. 8 weeks.	Y - Burnout Y - Resilience Y - Work engagement Y - Perceived stress	Perceived stress and burnout significantly decreased, and resilience and work engagement significantly increased after the intervention in the COVID cohort, showing that the online MIM was as effective as the face-to-face MIM (pre-COVID cohort).
Li et al. 2022 [61]	Online: Brief Mindfulness Meditation (JW2016 BMM). Lecture. Mindful breathing. 16 days. 15-min every day.	N - Depression N - Anxiety N - Perceived stress N - Insomnia	Significant decrease in post-test depression (*t* = 3.84, *p* < .001, *d* = .43) and insomnia (*t* = 2.27, *p* = .027, *d* = .28) (but not in anxiety and stress) in the IG. Significant decrease in post-test depression (but not in anxiety, stress, and insomnia) in the CG (*t* = 2.46, *p* = 0.021, *d* = 0.51). No significant differences in depression, anxiety, perceived stress, and insomnia between the IG and the CG.
Luton et al. 2021 [62]	Online platform, except the first session: Enhanced Stress-Resilience Training. Mindfulness-based exercises. Stress and coping techniques. 5 weeks. A 90-min initial session and 75-min tutorials.	N - Burnout Y - Stress Y - Mindfulness N - Depression N - Anxiety	Significant decreases in perceived stress (*p* < .010) and increases in mindfulness (*p* = .010) in the IG, while no significant changes in the CG. No statistical differences between both groups in burnout (*p* = .770) depression, and anxiety (*p* = .450).
Marotta et al. 2022 [63]	Face-to-face: Mindfulness-Based Stress Reduction (MBSR). Mindfulness meditation (breathing, eating, walking, etc.). Body-scan. Yoga. 8 weeks. 2 h every week. 30 min daily meditation practice at home.	Y - Psychological well-being Y - Perceived stress M - Burnout Y - Fear of COVID-19	Significant increase in psychological well-being (*p* = .0006) and depersonalization (*p* = .04), and significant decrease in perceived stress (*p* = .0001, emotional exhaustion (*p* = .0007), and fear of COVID-19 (*p* = .009) in the IG.
Miyoshi et al. 2022 [64]	Online: Mindfulness (breathing meditation, sitting meditation, body-scan). Yoga. 3 months. A weekly 1 h session.	N - Depression N – Burnout N - Stress N - Resilience M - Self-compassion N - Empathy	No significant changes were observed in depression, burnout, sense of coherence in the face of stress, resilience, self-compassion, and empathy. However, in self-compassion, the subscale common humanity increased significantly (5.6, 6.5, *p* < .05; *d* = .53), and over-identification decreased significantly (7.9, 6.7, *p* < .01; *d* = .91).
Nestor et al. 2023 [65]	In-person and remotely: Transcendental Meditation. Introduction. Verification of correct practice. Development of higher human potential and wellness. 12 weeks. 10 total sessions. 20 min practice twice a day. *3-month follow-up.*	Y - Depression Y - Anxiety Y - Insomnia M - Burnout Y - Well-being	Significant improvements in the IG at 1 and 3-month follow-up in anxiety (*p* < .001), depression (*p* = .001, .002), insomnia (*p* < .001), emotional exhaustion (*p* < .001), depersonalization (*p* = .01, < .001), and well-being (*p* < .001). The same results at post-intervention, except for depersonalization (no significant changes). CG outcomes did not change significantly from baseline, except for the 1 and 3-month follow-up of emotional exhaustion (*p* = .002, .04).
Nourian et al. 2021 [66]	Online: Meditations. Mindfulness. Yoga exercises. *Control group:* Music or training on caring for patients with COVID-19. 7 weeks.	M - Sleep quality	Sleep latency (*p* = .020) and subjective sleep quality (*p* = .000) were significantly higher in the IG. The total sleep quality scores and other subscales did not show any significant differences between the 2 groups.
Osman et al. 2021 [67]	Online (zoom): Mindfulness-based program. Three meditations. 3 min. breathing space. Discussions about the process. 4 weeks. 1 h every week.	Y - Trait mindfulness Y - Perceived stress Y - Burnout	Significant increase in trait mindfulness (*p* < .001) and personal accomplishment (*p* = .002). Significant decrease in perceived stress (*p* < .001) and emotional exhaustion (*p* = .04). No significant changes in depersonalization.
Pandey et al. 2021 [68]	Face-to-face: Postures (Asanas). Breathing regulation techniques (Pranayama). Meditation (Dhyana). 4 weeks. 24 sessions. 45 min.	Y - Quality of life	Significant change in physical domain (*p* = .001), psychological domain (*p* = .045) and in the total mean quality of life score (*p* = .028).
Prado et al. 2023 [69]	Mobile app: Synctuition. Binaural beats. Pure tones projected ED dichotically through headphones. 30 days. 20 to 30 min daily.	Y - Perceived stress	Significant decrease in perceived stress (*p* < .001).
Thimmapuram et al. 2021 [70]	Virtual, audio links: Heartfulness relaxation technique in the morning and for sleep. 4 weeks. 2 sessions a day. 6 min.	Y - Loneliness Y - Sleep quality	IG had a significant decrease in loneliness (*p* = .009) and increase in sleep quality (*p* = .001), compared to the CG.
Vajpeyee et al.2022 [71]	Mixed modality: Traditional yoga practices. Music in the form of standard instrumental music or music of participants’ choice. 1 month. Every day. 30 min.	Y - Depression Y - Anxiety Y - Stress	Significant positive impact of intervention in depression, anxiety, and stress, compared to the CG.
Yıldırım et al. 2022 [72]	Online (zoom): Mindfulness-based breathing (breath, body-scan, and emotions). Music therapy (light piano). *Control group:* relax in a quiet and calm setting for 30 min. 30 min in a single session.	Y - Anxiety Y - Psychological well-being Y - Work-related strain	IG had a significant decrease in anxiety (*p* < .001) and work-related strain (*p* < .001), and a significant increase in psychological well-being (*p* < .001), compared to the CG.

*IG*, intervention group; *CG*, control group; *Y*, yes, statistically significant effects of the MBI on the variable; *N*, no, non-statistically significant effects of the MBI on the variable; *M*, mixed, statistically significant effects of the MBI on some subscales of the variable or on some subgroup; *ND*, no data; *CI*, confidence interval; *p*, probability; *t*, Student *t* test; *d*, Cohen’s *d*, effect size; *Z*, Wilcoxon signed-rank test; *β*, beta coefficient

Sixteen studies focused their intervention specifically on mindfulness, understanding it as the use of different meditation strategies to achieve full awareness of the present moment. The tele-MBI carried out in the study by AlQarni et al. [45] included mindfulness of breath, movement, body sensations, emotions, thoughts, etc. Al Ozairi et al. [46] implemented a structured mindfulness meditation program adapted from the Mindfulness-Based Cognitive Therapy (MBCT), that incorporated breathing, sitting, sound, body-scan and walking meditations, and the promotion of compassion and kindness. The intervention performed in the study by Azizoddin et al. [47] was based on transcendental meditation and fundamentally consisted of the use of a repetitive mantra while the person is sitting with his/her eyes closed. The intervention carried out in the study by Franco and Christie [53] was an abbreviated adaptation for HCPs of the 8-week Mindful Self-Compassion (MSC) program developed by Neff and Germer [73]. The focus of the intervention was the development of self-compassion and included mindfulness, self-kindness, and the recognition of one’s common humanity. The 8-week Mindfulness Program, based on the foundations of the Mindfulness-Based Stress Reduction Program (MBSR), was applied by Gherardi-Donato et al. [54] and included different personal processes focused on breathing, the body, sensations, sounds, thoughts, and emotions. Hsieh et al. [55] applied a program based on gong meditation, so that the HCPs, lying on sleeping pads and covered with blankets, focused their attention on the sound of a gong with real-time adjusted rhythm. Ibrahim et al. [56] based their intervention on mindfulness breathing, a basic meditation technique focused on following the rhythm of breathing while inhaling and exhaling air. Headspace is the name of the mindfulness practice used in the study by Keng et al. [57], which includes introduction to mindfulness, mindful breathing, mindfulness of thoughts, and mindfulness of sounds, among others. Kim et al. [58, 59] applied a skill-based mindfulness program, called the Mindfulness Ambassador Program (MAP), which included mindful breathing, mindful listening, mind–body awareness, and paying attention and connecting authentically. Li et al. [61] applied a Brief Mindfulness Meditation (BMM) program, mainly focused on mindfulness breathing. Marotta et al. [63] used the MBSR [27], including mindfulness meditation, body awareness, and deepening behavior, thinking, feeling, and action. Nestor et al. [65] applied in their study a transcendental meditation technique. Osman et al. [67] analyzed the efficacy of the MBCT-4, a brief mindfulness program that includes meditations and breathing exercises. Prado et al. [69] used a mobile mindful meditation application that applies binaural beats (pure sounds, tones, and frequencies) dichotically through headphones. Thimmapuram et al. [70] evaluated the efficacy of heartfulness meditation, which includes heartfulness relaxation practice for meditation in the morning and heartfulness relaxation practice prior to sleep. The technique includes the conscious body scan and finishes by bringing our attention to the heart and the sensation of a light source emanating from it.

The other 12 studies combined mindfulness techniques with other types of interventions. Cao et al. [48] combined in their program Balint groups (meeting and support groups where they shared experiences, difficulties, emotions, thoughts, and proposed solutions) with MBSR (breathing, body scan, emotions, thoughts, loving-kindness, contemplation…). Cepeda-López et al. [49] conducted a mind-body-based intervention that included mindfulness-based stress reduction, single-focus meditation, self-regulation exercises (i.e., yoga qigong), breathing practices (i.e., diaphragmatic breathing), awareness practices, spirituality, and reframing strategies based on existential positive psychology (acceptance, letting it go…). DeTore et al. [50] included didactic information, experiential exercises, and testimonials from HCPs on resilience, mindfulness, and self-compassion in their intervention, based on cognitive-behavioral and mentalization techniques. Throughout the process, emphasis was placed on the implementation of this knowledge and skills in daily life, delving into the specific challenges that HCPs face during the COVID-19 pandemic. In the study carried out by Divya et al. [51], Sudarshan Kriya Yoga (SKY) was used, a method that combines controlled cyclical breathing and meditation, and which has its roots in traditional yoga. It includes awareness of breathing and controlled breathing with slow, normal, and fast rhythms. Fiol-DeRoque et al. [52] implemented a self-managed psychoeducational intervention focused on four fundamental areas: emotional skills, healthy lifestyle behavior, work stress and burnout, and social support, all based on cognitive-behavioral and mindfulness techniques. Mindfulness in Motion (MIM) is the name of the program developed in the research carried out by Klatt et al. [60], consisting of a mindfulness-based intervention that includes experiential mindfulness meditation and gentle yoga sessions. The Enhanced Stress-Resilience Training (ESRT) evaluated by Luton et al. [62] included mindfulness-based exercises, in addition to other techniques to deal with stress and burnout. The program carried out by Miyoshi et al. [64] combines mindfulness (sitting meditation, breathing meditation, body scan) and yoga exercises. Nourian et al. [66] used an online adaptation of the MBSR [25, 26]. The program included information on the nature of mindfulness, meditation practices, and yoga exercises. The protocol applied in the study by Pandey et al. [68] included yoga postures (Asanas), breathing regulation techniques (Pranayama), and meditation (Dhyana), which favor the achievement of a state of dissociation between oneself and disturbing thought or activities. The intervention carried out by Vajpeyee et al. [71] combined yoga sessions (which include meditation and deep breathing exercises) and music sessions (the participants could choose the type of music they preferred, and/or listen to instrumental music by Pandit VM Bhatt). Finally, Yildirim et al. [72] combined mindfulness-based breathing (focus on the breaths, on each part of the body, and on the emotions) and music therapy (light piano music as background music).

Regarding the modality of implementation, 4 interventions were carried out in-person through face-to-face sessions [53, 55, 63, 68], 20 in virtual/online format (through apps, WhatsApp, video files, audio files, reading files, tutorials, and/or phone conversations) [45, 46, 48–52, 54, 56–61, 64, 66, 67, 69, 70, 72], and 4 in a mixed modality [47, 62, 65, 71].

Regarding program integrity, it is worth highlighting that there is a great diversity of options. Thirteen of the interventions were conducted by expert and experienced trainers in mindfulness [47, 49, 51, 55, 58–60, 63, 65, 67, 70–72]. Five were carried out by health professionals (psychologists, psychiatrists, nurses, and doctoral level clinicians) experts in mindfulness [46, 48, 50, 52, 54]. At this point, it should be noted that, in the study by DeTore et al. [50], testimonials of HCPs about their experiences during the pandemic and their use of the skills learned in previous courses were also included. Two were developed by the researchers themselves [56, 62]. Two by the researchers together with psychologists and experienced trainers [64, 66]. And 6 of the studies do not indicate who prepared and/or carried out the intervention [45, 53, 57, 61, 68, 69].

Of all the studies, only 9 [47, 49–51, 53, 57–59, 65] included follow-up evaluations.

Finally, none of the studies reported adverse events related to MBIs.

In summary, there is a predominance of MBIs focused specifically on mindfulness (16/28), and in which meditations focus mainly on breathing, the body (body scan, body sensations, body awareness, heartfulness meditation), sounds (ambient sounds, gong meditation, binaural beats), stillness and movement (sitting and walking meditations), emotions and thoughts, and promoting compassion, self-compassion, kindness, and self-kindness. The rest of MBIs (12/28) combine mindfulness with other interventions, such as psychoeducation, cognitive-behavioral and mentalization techniques, emotional skills, coping skills, social support, yoga, diaphragmatic or controlled cyclical breathing, and music therapy. In general, there is a higher proportion of MBIs in virtual/online format (20/28), led by expert mindfulness trainers (13/22, taking into account that 6 papers did not specify who conducted the MBI), and without follow-up evaluations (19/28).

### Outcome measures and instruments

The mental health variables analyzed in the studies were, from highest to lowest frequency, stress, burnout, anxiety, depression, sleep quality, resilience, mindfulness, mental well-being, fear of COVID-19, compassion, compassion satisfaction, self-compassion, loneliness, post-traumatic stress, work engagement, self-efficacy, satisfaction with life, quality of life, and empathy (see Table 3).

- *Stress.* The stress perceived by HCPs has been evaluated in 17 of the studies using different versions of the Perceived Stress Scale (PSS) [47–49, 54, 55, 60–63, 67, 69], different versions of the Depression, Anxiety and Stress Scale (DASS) [51–53, 71], the Work-Related Strain Inventory (WRSI) [72], and the Sense of Coherence Scale (SOC-13) [64].
- *Burnout.* This variable has been evaluated in 15 of the studies through the Maslach Burnout Inventory (MBI) in different versions [47, 48, 50, 52, 59, 60, 62–65, 67], the Burnout Clinical Subtypes Questionnaire (BCSQ-12) [49], the Occupational Burnout Inventory (OBI) [55], and the Professional Quality of Life Scale (ProQOL) [53, 57].
- *Anxiety.* Anxiety levels in HCPs were analyzed in 14 papers using the Generalized Anxiety Disorder 7-item scale (GAD-7) [46, 47, 61], the Patient Health Questionnaire-4 (PHQ-4) [50], different versions of the Depression, Anxiety and Stress Scale (DASS) [51–53, 57, 71], different versions of the State-Trait Anxiety Inventory (STAI) [45, 62, 72], the Beck Anxiety Inventory (BAI) [54], and the Brief Symptom Inventory 18 (BSI-18) [65].
- *Depression.* The presence of depressive symptoms was analyzed in 13 of the studies using different versions of the Patient Health Questionnaire (PHQ) [46, 47, 50, 61, 62, 64], different versions of the Depression, Anxiety and Stress Scale (DASS) [51–53, 57, 71], the Beck Depression Inventory-II (BDI-II) [54], and the Brief Symptom Inventory 18 (BSI-18) [65].
- *Sleep quality.* This variable was analyzed in 8 of the studies through the Patient-Reported Outcomes Measurement Information System (PROMIS) Sleep Disturbance 8-item measure [47], the Pittsburgh Sleep Quality Index (PSQI) [51, 57, 66, 70], the Insomnia Severity Index (ISI) [52, 65], and the Athens Insomnia Scale (AIS) [61].
- *Resilience.* To evaluate, in 8 of the studies, this variable, the Brief Resilience Scale (BRS) [49, 50], the Connor-Davidson Resilience Scale (CD-RISC) in different versions [45, 51, 60, 64], the Short Resilience Survey (SRS) [53], and the Nicholson McBride Resilience Questionnaire (NMRQ) [58] were used.
- *Mindfulness.* This variable was the object of study in 8 of the articles, being measured through the Cognitive and Affective Mindfulness Scale (CAMS) in different versions [53, 62], the Five Facet Mindfulness Questionnaire in different versions [46, 54, 57], and the Mindful Attention Awareness Scale (MAAS) [49, 54, 67].
- *Mental well-being.* This variable was assessed in 8 studies using the World Health Organization-Five Well-Being Index (WHO-5) [45], the Ryff’s Psychological Well-Being Scale (SPWB) [48], the Warwick-Edinburgh Mental Well-being Scale (WEMWBS) (in a different version) [56, 65], the Personal Wellbeing Index (PWI) [57], the Psychological General Well-Being Index (PGWBI) [63], the Psychological Well-Being Scale (PWS) [72], and the PERMA Profiler [49].

Other variables were also analyzed, albeit in a smaller number of articles. Thus, *fear of COVID-19* was assessed in 2 papers by the Fear of COVID-19 Scale (FCV-19S) [57, 63]; the Compassion Scale (CS) was used in 1 study [53] to assess *compassion*; the Professional Quality of Life Scale (ProQOL) was used in 2 studies [53, 57] to assess *compassion satisfaction*; 4 studies evaluated *self-compassion* using the Self-Compassion Scale (SCS) [50, 53, 57, 64]; the feeling of *loneliness* and social isolation was evaluated in 2 of the studies using the UCLA Loneliness Scale [50, 70]; *post-traumatic stress* in 3 studies through the Davidson Trauma Scale (DTS) [52], the Professional Quality of Life Scale (ProQOL) [53], and the Posttraumatic Stress Disorder Checklist–Civilian Version (PCL-C) [57]; *work engagement* in 2 of the studies using the Utrecht Work Engagement Scale (UWES) [60] and the Job Engagement Scale (JES) [53]; *self-efficacy* in 1 article through the General Self-Efficacy Scale (GSE) [52]; *satisfaction with life* in 1 study through the Satisfaction With Life Scale (SWLS) [51]; *quality of life* in 1 article through the WHOQOL-BREF–survey form [68], and *empathy* of the HCPs in 1 study through the Jefferson Scale of Empathy (JSE) [64].

A great variability is observed in the number of variables analyzed in each study, from those that evaluate only 1 variable (4/28) to those that evaluate 11 variables (1/28).

Finally, of the 19 mental health variables evaluated in the studies, the most studied were stress, burnout, anxiety, depression, sleep quality, resilience, mindfulness, and mental well-being.

### MBIs effectiveness

The effects of the MBIs on each of the variables considered in the studies included in the systematic review are presented below (see Table 5 and Additional files 5 and 6):

- *Stress.* In most of the studies that included CGs and analyzed this variable (7/9), the MBIs showed to be effective in reducing stress at the end of the intervention [48, 53, 55, 62, 63, 71, 72], and 3 months post-intervention [53], compared to the CGs, in which no significant pre-post differences were observed. However, the study by Li et al. [61] did not find differences between both groups. Additionally, in the study by Fiol-De Roque et al. [52], a significant reduction in stress was observed at post-intervention only among HCPs receiving, in addition to the intervention, psychotherapy, or psychotropic medications, compared to the CG. In the case of most of the studies that did not include a CG (single-arm cohort) (7/8), the MBIs produced a statistically significant reduction in stress at the end of training [47, 51, 54, 60, 67, 69], at the 3-month follow-up [47], and at the 6-month follow-up [49]. However, in one of the studies, this reduction was not maintained 40 days later [51], and in another study [64], no differences pre-post intervention was found.
- *Burnout*. If the focus is on studies that included a CG, the MBIs applied in some of the studies (3/8) showed to be effective in reducing global burnout at post-intervention [48, 53, 55] and at a 3-month follow-up [53] in favor of the IGs. The study by Marotta et al. [63] found significant differences in favor of the IG in the emotional exhaustion scale, and Nestor et al. [65] in the same scale and in depersonalization. In other studies, the differences between groups were not significant at the end of the training [52, 57, 62] and 1 1 later [57]. No information on differences between groups in burnout was included in the study by DeTore et al. [50]. In most studies that did not include a CG (4/6), the interventions produced a significant reduction in this variable. Thus, the MBI applied by Azizoddin et al. [47] caused a significant reduction in burnout at the end of training and at the 3-month follow-up. Klatt et al. [60] also observed a decrease in burnout after the intervention. Kim et al. [59] showed a significant reduction in emotional exhaustion and depersonalization at the end of the intervention and at the 1-month follow-up. And Osman et al. [67] observed a significant reduction in emotional exhaustion and a significant increase in personal accomplishment. However, neither Cepeda-López et al. [49] nor Miyoshi et al. [64] found significant differences pre-post intervention in this variable.
- *Anxiety*. As a result of the MBIs, HCPs in half of the IGs (5/10) exhibited significant decreases from the pre- to post-intervention [50, 53, 71, 72], 1 month later [65], 2 months later [50] and 3 months later [53, 65] compared to the CGs, in which no significant decreases were observed. In the case of the MBI applied in the study by Fiol-DeRoque et al. [52], and as occurred in other variables, the reduction in anxiety was also significant at post-intervention, but only in the case of the HCPs who received the intervention and who were also receiving psychotherapy or psychopharmacological treatment. AlQarni et al. [45] observed a significant reduction in state anxiety in both MBI and relaxation groups after the intervention, with no significant differences between them. In other cases, no significant pre-post-intervention differences were found between both groups at the end of the intervention [57, 61, 62] or at 1-month follow-up [57]. Regarding the studies that did not include a CG, in all the cases (4/4) there were significant decreases in anxiety symptoms immediately after finishing the program [46, 47, 51, 54] and at the 3-month follow-up [47]. Only in the study by Divya et al. [51] this reduction was not maintained 40 days after the MBI.
- *Depression.* The intervention applied in a few of the studies that included a CG (3/8) showed to be effective in reducing depression. The MBI applied by DeTore et al. [50] caused a significant decrease in depressive symptoms at post-intervention and at a 2-month follow-up, compared to the CG. Vajpeyee et al. [71] found these same results at post-intervention, and so did Nestor et al. [65] at 1- and 3-month follow-ups. Other studies did not find significant differences between groups at the end of the training [52, 53, 57, 61, 62], 1 month later [57], or 3 months later [53]. In the case of studies that did not include a CG, most of them (4/5) showed significant reductions in this variable at post-intervention. Even though Al Ozairi et al. [46], Azizoddin et al. [47], Divya et al. [51], and Gherardi-Donato et al. [54] observed a significant reduction in depressive symptoms at post-intervention due to MBIs, this reduction was still maintained at the 3-month follow-up in the second study, while it was not maintained 40 days later in the third. In the study conducted by Miyoshi et al. [64], no significant differences in the pre-post intervention were found in this variable.
- *Sleep quality*. Some studies (2/6) indicate significant improvements in this variable. The study by Thimmapuram et al. [70] showed significant improvements after the MBI and compared to the CG. The same results were found by Nestor et al. [65] at 1- and 3-month follow-up. Other studies show confusing results. Keng et al. [57] found significant improvements at a 1-month follow-up, but not at the end of the intervention, where no differences were observed. Nourian et al. [[66] used the same instrument (PSQI) and analyzed the differences not only in the total score, but also in the different subscales, finding that, after the MBI, subjective sleep quality and sleep latency were significantly higher in the IG, but not the total sleep quality score and other subscales scores. Fiol-DeRoque et al. [52] found significant improvements in sleep quality at post-intervention only in the intervention subgroup that was also receiving psychotherapy or psychotropic medications. Li et al. [61] did not find significant differences between groups. Significant improvements were observed in this variable in the studies with a single-arm cohort (2/2). The sleep quality of the HCPs improved significantly immediately after the MBIs [47, 51] and 3 months later [[47], except for the absence of significant changes pre-40 days post in the study by Divya et al. [51].
- *Resilience*. In the case of studies that included CGs (3 studies), the results were confusing. AlQarni et al. [45] did not observe significant improvements in resilience after the intervention neither in the MBI group nor in the relaxation group. DeTore et al. [50] did not find significant changes in the IG from baseline to post-intervention, but they did show a significant increase from baseline to 2 months following the course, not observing this increase in the CG. Franco and Christie [53] also reported similar results, but only in the resilience-decompression component, in which there was evidence of a significant increase at the 3-month follow-up only (but not at post-intervention) when compared to the CG. In the resilience-activation component, no significant differences were observed between groups. Regarding the studies with single-arm cohorts, some of them (3/5) showed significant improvements in resilience at post-intervention [51, 58, 60], 1 month later [58], and 40 days later [51]. But in one of the studies [64], no significant differences were found from pre- to post-intervention, and another study [49] even showed a significant decrease in this variable at 6-month follow-up.
- *Mindfulness.* In all the studies (4/4), HCPs in the IGs exhibited significant increases in this variable due to the MBIs, compared to the CGs, at post-intervention [48, 53, 62], at the 1-month follow-up [57], and at the 3-month follow-up [53]. Only Keng et al. [57] found no differences between groups at the end of the intervention. In all the studies with one single-arm (4/4), the HCPs showed significant positive effects on mindfulness at post-intervention [46, 54, 67], and at 6-month follow-up [49].
- *Mental well-being.* Most studies (5/7) reported significant improvements in this variable due to the MBIs in the IGs compared to the CGs [48, 56, 63, 65, 72]. One study [45] observed significant improvements in both groups (MBI and relaxation), with a higher improvement in the MBI group. Keng et al. [57] did not find differences between groups neither at post-intervention nor at the 1-month follow-up. In the single-arm study by Cepeda-López et al. [49], subjective well-being significantly decreased from pre-test to 6-month follow-up.

Regarding the rest of the variables, participants in the IG in the study by Keng et al. [57] reported significant decreases in *fear of COVID-19* at the 1-month follow-up compared to the CG, but no differences between groups were observed immediately after finishing the intervention. Instead, Marotta et al. [63] did observe that the fear of COVID-19 was significantly reduced in the IG at the end of the intervention, unlike the CG, in which there were no significant changes. The study by Franco and Christie [53] did not find differences in the levels of *compassion* between groups at post-intervention, but it did at the 3-month follow-up, in favor of a significant increase in the IG. No differences were found in *compassion satisfaction* and *self-compassion* between groups at the end of the training [57, 64], but were found after 2 weeks [53], 1 month [57], and 3 months [53]. It is worth mentioning that in the study carried out by Miyoshi et al. [64], although the global self-compassion score did not improve after the intervention, the scores on the Common humanity and Overidentification scales did. No data was provided on the results in this variable in the study carried out by De Tore et al. [50]. Thimmapuram et al. [70] reported significant decreases in the *loneliness* scores in the IG, unlike the CG, but no information about changes in this variable due to the intervention is included in the study by DeTore et al. [50]. Two studies found no significant differences in *post-traumatic stress* between groups at the end of the training [53, 57], 1 month later [57], and 3 months later [53]. Fiol-DeRoque et al. [52] only observed a significant reduction in this variable at post-intervention in the IG receiving psychotropic medications. Regarding *work engagement*, Franco and Christie [53] found no significant differences between groups, neither at post-intervention nor 3 months later, while Klatt et al. [60] concluded that it increased due to the intervention. Fiol-DeRoque et al. [52] found no significant differences in *self-efficacy* between groups. The one single-arm study of Divya et al. [51] showed a statistically significant improvement of *satisfaction with life* in HCPs immediately after the program, and it continued to increase on day 40. The one single-arm study of Pandey et al. [68] showed a significant statistical improvement in the total score of *quality of life* due to the MBI. Finally, no significant differences in pre-post intervention were observed in *empathy* in the study of Miyoshi et al. [64].

In summary, of the 8 mental health variables most evaluated in all studies, MBIs have shown the greatest evidence of effectiveness in (yes-mixed/total): stress (13-2/17), mindfulness (7-1/8), and mental well-being (6-0/8). In the other 5 variables, although significant effects have been observed due to the MBIs, the results are not so conclusive: burnout (7-2/15), anxiety (8-3/14), depression (6-1/13), sleep quality (3-4/8), and resilience (3-2/8). If only the RCTs are considered, the results are similar: stress (5-1/7), mindfulness (1-1/2), mental well-being (4-0/5), burnout (2-2/5), anxiety (2-2/6), depression (1-1/4), sleep quality (1-3/5), and resilience (0-0/1) (see Additional files 5 and 6). It should also be added that, when the modality of implementation is considered, if all the studies and all areas of mental health are considered, a greater proportion of effectiveness is observed for MBIs carried out face-to-face and/or in a mixed modality (26-4/36), than those carried out online (34-17/72).

## Discussion

The general objective of this systematic review has been to analyze the studies on MBIs aimed at HCPs during the COVID-19 pandemic, to evaluate their content and their effectiveness in different variables related to mental health. Considering when the pandemic began, only studies published in the last 4 years have been included in the review.

The methodological quality of the 11 RCTs is, in general, satisfactory, observing a prevalence of low risk in the different bias domains, although the presence of high and unclear risk in certain domains (blinding participants and researchers, random sequence generation, and allocation concealment) should be highlighted. As for the methodological quality of the 17 NRCTs and NRNCTs, most of them show some high or unclear risk of bias. Some studies include small sample sizes, and only 5 analyze an alternative treatment group or active CG. In addition, in many of the studies, the samples were self-selected, which may influence the results obtained due to the level of motivation of the participants. Therefore, the results of the studies included in the review should be assessed with caution, as their overall robustness is moderate. RCTs with a higher methodological quality would be necessary to reach more compelling conclusions in this context.

The studies include different types of mindfulness, such as transcendental meditation, body scan, mindfulness focused on breathing, mindfulness of thoughts, mindfulness of sounds, compassion, self-compassion, or heartfulness meditation. Although more than half of the studies focus their intervention on mindfulness as the only strategy, the rest of them combine mindfulness with yoga exercises, mentalization, music, and/or other cognitive-behavioral interventions (emotional skills, coping skills, healthy lifestyle behavior, etc.). This diversity may have influenced the indicators of mental health, so caution should be exercised when generalizing the results.

The need for social distancing caused by the COVID-19 pandemic has made it necessary to virtualize interventions, which would explain the greater prevalence of these MBIs found in the studies. It is important to note that one of them compares whether virtual intervention (during the COVID-19 pandemic) is as effective as in-person intervention (before the COVID-19 pandemic), finding evidence in this regard [60]. However, if the results of the studies included in the review are compared, a greater proportion of effectiveness is observed in MBIs carried out in person and/or in a mixed format than those that have been applied in a virtual modality. A possible explanation for these results is the positive effect that human presence and contact have on any type of intervention, especially considering the situation of social isolation experienced during the COVID-19 pandemic.

Regarding the integrity of the programs, interventions are predominantly carried out by expert instructors, but some have also been implemented by health professionals or by researchers. This diversity may also have had effects on the results obtained.

The most evaluated variables in the reviewed studies have been stress, burnout, anxiety, and depression. To a lesser extent, the efficacy of MBIs on sleep quality, resilience, mindfulness, and mental well-being has been analyzed. The levels of fear of COVID-19, compassion, compassion satisfaction, self-compassion, loneliness, post-traumatic stress, work engagement, self-efficacy, satisfaction with life, quality of life, and empathy have also been studied, but only in some of the studies. The interest in all these indicators of mental health has also been frequent in other studies on the efficacy of MBIs for HCPs before the COVID-19 pandemic [74–76].

The most widely used assessment instruments in the studies have been the Perceived Stress Scale (PSS), the Maslach Burnout Inventory (MBI), and the Depression, Anxiety and Stress Scale (DASS), in different versions, which have also been among the most used in pre-pandemic studies [20, 77]. Apart from these, a great variability of outcome measures has been observed, which can make it difficult to compare results between studies that evaluate the same psychological variables. By examining this large variability, a core set of variables can be extracted which are the most frequently studied, namely stress, burnout, and symptoms of anxiety and depression, mainly assessment with the PSS, the MBI, and the DASS. This set of assessments responds to the essential aspects of the evaluation of mental health in an occupational context.

The mental health variables in which MBIs have shown greater effectiveness are stress, mindfulness, and mental well-being. Most of the studies included in the review show that MBIs produce a significant reduction in the stress levels of the HCPs. These results are consistent with those of other studies carried out before the COVID-19 pandemic [17, 18, 20]. These data show that, in a situation of such tension, overload, and uncertainty as the COVID-19 pandemic, MBIs are effective in relieving the stress experienced by HCPs. The levels of *mindfulness* increased in all the studies that evaluated it, in the same line as previous papers [74, 78]. These results are usually indicative of being more present and not being in an “autopilot state” [67]. Furthermore, most of the studies show improvements in *mental well-being*, as in pre-COVID-19 research [15]. In this sense, it could be stated that MBIs have contributed to increasing the subjective feeling of emotional well-being of HCPs, despite experiencing an extreme situation in the work context.

Some evidence has also been found about the effectiveness of MBIs in improving levels of *burnout, anxiety, depression, sleep, and resilience*, but the data are not as conclusive as in the case of the previously mentioned variables. These results are striking, as most studies prior to the pandemic showed the benefits of MBIs at the levels of these areas of mental health in HCPs [20, 21, 74, 78–82]. One possible explanation for these results is the great influence that the COVID-19 pandemic had on the mood and emotional balance of HCPs. We should not forget that the first waves of the pandemic meant for many HCPs an increase in shifts and work demands, fear of infecting themselves and their loved ones, and witnessing illness and/or death due to COVID-19, which could have caused them great physical and mental overload. This could explain why the MBIs were not powerful enough to reflect improvements in these other areas of mental health.

Regarding the least evaluated variables, there are significant improvements after the MBIs in *loneliness, satisfaction with life,* and *quality of life*; results both for and against the influence of MBIs on *fear of COVID-19, compassion, compassion satisfaction, self-compassion, post-traumatic stress,* and *work engagement*; and no significant differences in *self-efficacy* and *empathy.* It is not possible to draw conclusions regarding any of these variables, as each of them has only been researched in a small number of studies; therefore, it would be risky to make comparisons with previous research.

It could be concluded that the most powerful results are those referring to the reduction of stress and the improvement of mindfulness and mental well-being, after the application of the MBIs, of the HCPs who were working on the front lines during the COVID-19 pandemic.

In general, in an exceptional situation of high workload for HCPs, MBIs have had moderately good results. Naturally, these results also depend on the fact that any interventions focused on HCPs have been well received during the COVID-19 pandemic. Now it is a matter of learning from these experiences to be able to design effective interventions in the future.

The results can be described as promising but tentative; therefore, it is necessary that future research includes RCTs or robust study designs, large sample sizes, not only passive but also active CGs, and medium to long-term follow-up evaluations to check whether the effects of MBIs are maintained over time. It would also be desirable to analyze the results according to different types of HCPs (doctors, nurses, physiotherapists, psychologists, occupational therapists, etc.), since in most of the studies included in this systematic review these professionals were mixed within the samples.

As there are interventions that only include mindfulness and others that also include other techniques, it is difficult to demonstrate which is the active ingredient or mechanism of action in the case of combined interventions. It would be necessary to carry out research that compares the results, in the same psychological variables and with different samples, of interventions focused exclusively on mindfulness and interventions that also include other techniques. In addition, the homogeneity in the implementation of the interventions, by specialized professionals, would facilitate the comparison process. It could also be useful to identify the number of hours of mindfulness practice needed to start seeing effects. And it will also be useful in the future to distinguish between nonspecific effects, well-being in general, and specific effects related to the performance of professional tasks.

Furthermore, since virtual interventions facilitate access to a larger number of HCPs, save time, and are more cost-effective, it is necessary to evaluate whether they can continue to be maintained, even if health restrictions have been eliminated. Perhaps a mixed modality (online and in-person MBIs) is a good option, so as not to lose the benefits of face-to-face contact, an issue that also emerges from the results of this review.

Finally, it is important to identify and control moderating variables that may be influencing the results, such as whether the HCPs are also receiving psychotherapy and/or taking psychoactive drugs, whether they have prior training/experience in mindfulness, and whether, in addition to mindfulness sessions, they also practice meditation in their daily life, how often and for how long. Additionally, the reliability of the data would improve with the use of consistent outcome measures and momentary ecological assessment (a novel methodology that basically consists of evaluating at the moment, normally by electronic devices, and thus avoiding inference biases that occur in the traditional retrospective evaluation).

Considering the methodological limitations of the articles analyzed, it is important to highlight the MBIs that have been carried out, with the existing sanitary and human limitations, to contribute to the mental health of HCPs in such a difficult historical moment. Adhering to the maxim “do what you can with what you have,” not only a significant effort has been made to care for our caregivers during the COVID-19 pandemic, but also scientific data has been obtained that seems to demonstrate the usefulness of the MBIs in this population and during this health crisis, specially to alleviate stress and to improve mindfulness and well-being.

### Limitations

There are several limitations of the present study. Although databases of recognized prestige have been used as a search strategy, others such as Cochrane Central or CINAHL could also have been considered. Furthermore, the different modalities of MBIs have not been able to be grouped due to their heterogeneity. Not all studies have analyzed the same mental health variables, nor have they used the same standardized assessment instruments. The duration of the MBIs and the characteristics of the HCPs to whom the interventions have been applied are also highly variable between studies. This has made it unfeasible to carry out a meta-analysis, which would have been desirable to extract more robust and consistent results.

The most obvious strength of this review is that it focuses specifically on MBIs while being open to all types of HCPs, standardized assessment instruments and expected outcomes. This approach provides a rich and varied overview. It also provides a better insight into future research needs in this area.

## Conclusions

From the reviewed studies, it could be concluded that, although previous papers have shown the effectiveness of MBIs in a wide variety of areas of mental health of HCPs, the MBIs applied to HCPs who have been working on the front lines during the COVID-19 pandemic contributed to improving, mainly, their levels of stress, mindfulness, and emotional well-being. However, no conclusive results have been found regarding their effectiveness in other facets of mental health (such as burnout, anxiety, depression, sleep quality, resilience, and others).

Therefore, MBIs have been shown to be moderately effective in times of extreme tension and overload, health uncertainty, and lack of resources for the HCPs.

### Supplementary Information

Additional file 1: PRISMA 2020 Checklist

Additional file 2: Literature search strategy

Additional file 3: Data extraction form

Additional file 4: Criteria to identify bias

Additional file 5: Effectiveness of MBIs on the variables analyzed in the RCTs

Additional file 6: Effectiveness of MBIs on the variables analyzed in the NRCTs and NRNCTs

## Data Availability

Not applicable. All data generated or analyzed during this study are included in this article [and its supplementary information files].

## Abbreviations

COVID-19: Coronavirus disease 2019
HCPs: Health care professionals
MBIs: Mindfulness-based interventions
PRISMA: Preferred Reporting Items for Systematic reviews and Meta-Analyses
PICOS: Population-Intervention-Comparison-Outcome-Study design
CG: Control group
IG: Intervention group
RCT: Randomized controlled trial
NRCT: Non-randomized controlled trial
NRNCT: Non-randomized non-controlled trial
RACT: Randomized active controlled trial
MeSH: Medical Subject Headings
MBCT: Mindfulness-Based Cognitive Therapy
MBSR: Mindfulness-Based Stress Reduction Program
