# Supplementary material for: Mindfulness-based interventions for improving mental health of frontline healthcare professionals during the COVID-19 pandemic: a systematic review
Source: Syst Rev. 2024 Jun 20;13:160. doi: 10.1186/s13643-024-02574-5 (PMC11188518; doi:10.1186/s13643-024-02574-5)
Supplement: Supplementary file 2 — Additional file 2: Literature search strategy [file 13643_2024_2574_MOESM2_ESM.docx]

Additional file 2. Literature search strategy

| **Web of Science** |
| --- |
| (mindfulness OR mindful OR meditation OR breathing OR breath OR contemplative OR compassion) (Topic) and (health personnel OR medical staff OR nurses OR nursing staff OR physicians OR healthcare providers OR healthcare workers OR healthcare professionals) (Topic) and (COVID-19 OR SARS-CoV-2) (Topic) |
| **Scopus** |
| ( ( TITLE-ABS-KEY ( mindfulness OR mindful OR meditation OR breathing OR breath OR contemplative OR compassion ) AND TITLE-ABS-KEY ( health AND personnel OR medical AND staff OR nurses OR nursing staff OR physicians OR healthcare AND providers OR healthcare AND workers OR healthcare AND professionals ) AND TITLE-ABS-KEY ( covid-19 OR sars-cov-2 ) ) |
| **PUBMED** |
| ("Mindfulness"[MeSH] OR "Meditation"[MeSH] OR "Breathing Exercises"[MeSH] OR “Self-Compassion” [MeSH]) AND ("Health Personnel"[MeSH]) OR "Medical Staff"[MeSH] OR "Nurses"[MeSH] OR "Nursing Staff"[MeSH] OR "Physicians"[MeSH]) AND ("COVID-19"[MeSH]) OR "SARS-CoV-2"[MeSH]) |
| **PsycINFO** |
| ( mindfulness OR mindful OR meditation OR breathing OR breath OR contemplative OR compassion ) AND ( health personnel OR medical staff OR nurses OR nursing staff OR physicians OR healthcare providers OR healthcare workers OR healthcare professionals ) AND ( COVID-19 OR SARS-CoV-2 ) |
