# Supplementary material for: Mindfulness-based interventions for improving mental health of frontline healthcare professionals during the COVID-19 pandemic: a systematic review
Source: Syst Rev. 2024 Jun 20;13:160. doi: 10.1186/s13643-024-02574-5 (PMC11188518; doi:10.1186/s13643-024-02574-5)
Supplement: Supplementary file 3 — Additional file 3: Data extraction form [file 13643_2024_2574_MOESM3_ESM.docx]

Additional file 3. Data extraction form

| Reviewer |  |
| --- | --- |
| **General information** | |
| 1^st^ Author, Year |  |
| Title |  |
| Country |  |
| **Characteristics of included studies** | |
| Objective |  |
| Study design |  |
| Recruitment period |  |
| Setting |  |
| Population |  |
| Sample size |  |
| Sample characteristics |  |
| **Intervention characteristics** | |
| Intervention guiding theory |  |
| Modalidad de realización |  |
| Intervention Content |  |
| Timing of intervention (sessions number and length) |  |
| Duration of intervention |  |
| Time follow up |  |
| Outcomes and time points |  |
| **Intervention results** | |
| Findings |  |
| Author’s conclusion |  |
| Theory to explain the findings |  |
| Source of funding |  |
