# Supplementary material for: Mindfulness-based interventions for improving mental health of frontline healthcare professionals during the COVID-19 pandemic: a systematic review
Source: Syst Rev. 2024 Jun 20;13:160. doi: 10.1186/s13643-024-02574-5 (PMC11188518; doi:10.1186/s13643-024-02574-5)
Supplement: Supplementary file 4 — Additional file 4: Criteria to identify bias [file 13643_2024_2574_MOESM4_ESM.docx]

Additional file 4. Criteria to identify bias

| **1st Author, Year** | |  | | |
| --- | --- | --- | --- | --- |
| Bias domain | Source of bias | | Judgment | Support for judgment |
| Selection | Random sequence generation | | Low risk  Unclear risk  High risk |  |
|  | Allocation concealment | | Low risk  Unclear risk  High risk |  |
| Performance | Blinding participants and researchers | | Low risk  Unclear risk  High risk |  |
| Detection | Blinding of outcome assessment | | Low risk  Unclear risk  High risk |  |
| Attrition | Incomplete outcome data | | Low risk  Unclear risk  High risk |  |
| REporting | Selective reporting | | Low risk  Unclear risk  High risk |  |
| Other |  | |  |  |

The Cochrane Collaboration’s tool for assessing risk of bias in randomised trials (Higgins, 2011)

| **1^st^ Author, Year** |  | |
| --- | --- | --- |
| **Bias Domain** | Judgment | Support for judgment |
| **Pre-intervention** | | |
| Confounding | Low risk  Unclear risk  High risk |  |
| Selection of participants | Low risk  Unclear risk  High risk |  |
| **At intervention** | | |
| Classification of interventions | Low risk  Unclear risk  High risk |  |
| **Post-intervention** | | |
| Deviations from intended interventions | Low risk  Unclear risk  High risk |  |
| Missing data | Low risk  Unclear risk  High risk |  |
| Measurement of outcomes | Low risk  Unclear risk  High risk |  |
| Selection of the reported result | Low risk  Unclear risk  High risk |  |

ROBINS-I: a tool for assessing risk of bias in non-randomised studies of interventions (Sterne, 2016)
