# Supplementary material for: Mindfulness-based interventions for improving mental health of frontline healthcare professionals during the COVID-19 pandemic: a systematic review
Source: Syst Rev. 2024 Jun 20;13:160. doi: 10.1186/s13643-024-02574-5 (PMC11188518; doi:10.1186/s13643-024-02574-5)
Supplement: Supplementary file 6 — Additional file 6: Effectiveness of MBIs on the variables analyzed in the NRCTs and NRNCTs [file 13643_2024_2574_MOESM6_ESM.docx]

Additional file 6. Effectiveness of MBIs on the variables analyzed in the NRCTs and NRNCTs

| Variables | Al Ozairi | Azizoddin | Cepeda-López | DeTore | Divya | Franco | Gherardi-Donato | Ibrahim | Kim* | Klatt | Luton | Miyoshi | Nestor | Osman | Pandey | Prado |
| --- | --- | --- | --- | --- | --- | --- | --- | --- | --- | --- | --- | --- | --- | --- | --- | --- |
| Stress | - | Yes | Yes | - | Mixed | Yes | Yes | - | - | Yes | Yes | No | - | Yes | - | Yes |
| Burnout | - | Yes | No | No data | - | Yes | - | - | Yes | Yes | No | No | Mixed | Yes | - | - |
| Anxiety | Yes | Yes | - | Yes | Mixed | Yes | Yes | - | - | - | No | - | Yes | - | - | - |
| Depression | Yes | Yes | - | Yes | Mixed | No | Yes | - | - | - | No | No | Yes | - | - | - |
| Sleep quality | - | Yes | - | - | Mixed | - | - | - | - | - | - | - | Yes | - | - | - |
| Resilience | - | - | No | Mixed | Yes | Mixed | - | - | Yes | Yes | - | No | - | - | - | - |
| Mindfulness | Yes | - | Yes | - | - | Yes | Yes | - | - | - | Yes | - | - | Yes | - | - |
| Mental well-being | - | - | No | - | - | - | - | Yes | - | - | - | - | Yes | - | - | - |
| Fear of COVID-19 | - | - | - | - | - | - | - | - | - | - | - | - | - | - | - | - |
| Compassion | - | - | - | - | - | Mixed | - | - | - | - | - | - | - | - | - | - |
| Compassion satisfaction | - | - | - | - | - | Yes | - | - | - | - | - | - | - | - | - | - |
| Self- compassion | - | - | - | No data | - | Yes | - | - | - | - | - | Mixed | - | - | - | - |
| Loneliness | - | - | - | No data | - | - | - | - | - | - | - | - | - | - | - | - |
| Post-traumatic stress | - | - | - | - | - | No | - | - | - | - | - | - | - | - | - | - |
| Work-  engagement | - | - | - | - | - | No | - | - | - | Yes | - | - | - | - | - | - |
| Self-efficacy | - | - | - | - | - | - | - | - | - | - | - | - | - | - | - | - |
| Satisfaction with life | - | - | - | - | Yes | - | - | - | - | - | - | - | - | - | - | - |
| Quality of life | - | - | - | - | - | - | - | - | - | - | - | - | - | - | Yes | - |
| Empathy | - | - | - | - | - | - | - | - | - | - | - | No | - | - | - | - |

^*two studies; Yes: statistically significant effects of the MBI on the variable; No: non-statistically significant effects of the MBI on the variable. Mixed: statistically significant effects of the MBI on some subscales of the variable.^
